# A Noninvasive Five-Parameter Bedside Score for Predicting a Difficult Airway in the Emergency Department: A Prospective Observational Comparative Study

**DOI:** 10.7759/cureus.102515

**Published:** 2026-01-28

**Authors:** Anagani Hrushikesh, Chinnam Vishnupriya, Chetla Rakesh, Meesaraganda Chucharitha, Vanjavakam Sahithya, Sreekrishnan Trikkur, Gireesh Kumar

**Affiliations:** 1 Department of Emergency Medicine, Amrita Institute of Medical Sciences, Ernakulam, IND; 2 Hospital Administration, AIG Hospital, Hyderabad, IND; 3 Health Economic Outcomes Research, Jawaharlal Institute of Postgraduate Medical Education and Research, Puducherry, IND

**Keywords:** body mass index: bmi, difficult airway management, emergency departments, novel scoring system, prediction of difficult airway

## Abstract

Introduction: Airway management is a critical and challenging component of emergency care, requiring skilled interventions to ensure safe intubation, particularly in difficult airway scenarios. Early identification is essential to minimize intubation attempts and reduce major airway-related complications. Although several assessment tools and scoring systems have been proposed, their effectiveness as unstructured individual predictors remains uncertain. This study aimed to develop a simple scoring system for predicting difficult airways in the emergency department (ED).

Aim: To develop and preliminarily validate an innovative, easy-to-use, and uncomplicated scoring system for predicting the difficult airway in the ED.

Methodology: This prospective observational study was conducted in the ED of a tertiary medical center in India (January 2023-August 2025) and included 200 conscious adult patients. Five bedside predictors (body mass index (BMI), retrognathia, upper lip bite test (ULBT), thyromental distance (TMD), and hyomental distance) were assessed to develop a composite score for difficult airway prediction. The Modified Mallampati classification (MMC) (Class 3/4) was used as the reference standard for initial validation prior to assessment against the Cormack-Lehane classification. Diagnostic performance of individual predictors and the composite score was evaluated using sensitivity, specificity, predictive values, and receiver operating characteristic (ROC) analysis.

Results: Among 200 patients (n=200), 82 (n=82) were classified with a MMC of 3/4, resulting in a 41% incidence (n=82) of difficult airways. Among individual parameters, BMI >30 kg/m² demonstrated the highest individual sensitivity (79.3%), while the ULBT showed the highest specificity (94.9%). The novel scoring system achieved an overall sensitivity of 79.27% and a specificity of 71.2%. The short TMD was the most frequently observed positive individual predictor (n=59). The McNemar test indicated a statistically significant difference in the proportions of discordant classifications between the new score and the MMC (p=0.006), suggesting they do not classify patients identically. However, the kappa coefficient of 0.522 demonstrated a moderate level of overall agreement. ROC analysis demonstrated good discriminative ability (AUC=0.752, 95% CI: 0.683-0.822, p<0.001), validating the score’s diagnostic strength.

Conclusion: This study presents a tentative confirmation of a concise, five-parameter bedside scoring tool that displays strong discriminative ability in predicting a challenging airway in the ED environment. The score outperforms individual predictors and the MMC when used on its own by synthesizing predictable clinical and anatomical variables that can be obtained in a short period of time. Its high negative predictive value makes it easy to rule out airway difficulty early and to proactively deal with airway management. To verify the applicability and clinical relevance of the suggested tool, multicenter validation is necessary.

## Introduction

Airway management in an emergency department (ED) setting is the most crucial and complex task for emergency physicians [1]. Adept and immediate intervention is required to ensure airway management. Compared to elective intubation in a controlled operating room setting, emergency intubation in the ED is more challenging due to the unpredictable condition of acutely ill patients [2]. Multiple attempts at intubation increase the risk of complications like cardiac arrest, arrhythmia, regurgitation, and airway trauma [3]. Hence, an early identification of difficult intubation is crucial for prompt detection, adequate preparation for further management, and preventing intubation-associated complications.

Any clinical scenario involving anticipated or unanticipated difficulty or failure experienced by a physician skilled in anesthesia care is considered a difficult airway. This includes, but is not restricted to, any of the following: facemask ventilation, laryngoscopy, ventilation using a supraglottic airway placement, tracheal intubation, extubation, or emergency invasive airway access [4]. A failed airway is defined as “a clinical situation when a skilled physician makes three unsuccessful attempts at intubation” [5]. Various literature mentions the definition of a difficult airway, but within relatively general limits. The American Society of Anesthesiologists Task Force on Management of the Difficult Airway defines a difficult airway as a clinical situation where a qualified anesthesiologist experiences difficulties with face mask ventilation of the upper airway, tracheal intubation, or both [4,6]. The term difficult may be defined in various ways, including challenges with face mask ventilation, laryngoscopy, tracheal intubation, or failed intubation [6]. In literature relevant to emergency medicine, the difficult airway is most often categorized as difficult mask ventilation, difficult intubation, and difficult cricothyroidotomy (when performed by skilled personnel). A difficult or failed airway does not have a universal and sole definition [3-6].

Traditionally, a wide range of scoring systems and assessment tools have been utilized to forecast airway difficulty. Among these, the Modified Mallampati classification (MMC) remains among the most widely recognized and employed methods [4,7,8]. It evaluates the visibility of the oropharyngeal structures during mouth opening and has been instrumental in stratifying patients based on anticipated intubation challenges. Despite its prevalence, the MMC is not without limitations, particularly in diverse patient populations and various clinical settings. Its reliance on visual inspection and subjective interpretation can sometimes lead to variability in predictive accuracy [9].

In addition to the Mallampati grading (MPG), other assessments such as the Wilson score (WS), MMC, Modified Mallampati Assessment Classification (MTAC), and Simplified Airway Risk Index (SARI) have been developed to enhance predictive capabilities [4,9,10]. However, these tools often involve complex procedures or multiple variables that can complicate their application in fast-paced emergency scenarios. The effectiveness of combining multiple unstructured tests remains an area of ongoing investigation, as the integration of these assessments into a cohesive and practical prediction model is challenging. Before performing intubation, assessing the airway for any potential challenges that could hinder intubation or effective oxygenation using bag-mask ventilation is crucial. Failure to conduct a thorough evaluation may result in a "cannot intubate, cannot oxygenate (CICO)" scenario, potentially causing significant harm or death to the patient [4,6]. Therefore, it is advised that predictors of difficult airways be carefully considered before initiating emergency airway management [6].

Though various tools like SARI, LEMON (Look, Evaluate 3-3-2 rule, Mallampati class, Obstruction, Neck Mobility), WS, and MTAC scores have been proposed to predict difficult airways, it is still unclear whether relying on multiple unstructured tests is less reliable than using well-defined scoring systems [4,11]. Emergency healthcare professionals must be trained in the systematic evaluation of difficult airway components, including difficult facemask ventilation, difficult laryngoscopy, and difficult supraglottic airway device placement, as these factors collectively contribute to the risk of CICO scenarios [4,11].

Hence, this prospective observational study aimed to establish an easy, innovative, and uncomplicated score by using simple, non-invasive tests to predict difficult airways in the ED.

## Materials and methods

Study design

This research was conducted as a prospective, observational, and comparative analysis of our novel predictive score against the established MMC in predicting difficult airways. The study was conducted in the Department of Emergency Medicine of a tertiary medical center from January 2023 to August 2025. The tertiary care center offered a diverse population suitable for the study. The study was approved by the Institutional Review Board and Ethics Committee of Amrita School of Medicine (EC/NEW/INST/2023/KL/0379), as no ethical or scientific concerns were involved. Participants were included in the study after providing verbal and signed informed consent.

Study participants

Study participants were recruited according to the inclusion and exclusion criteria. The study participant inclusion and exclusion criteria are mentioned in Table 1.

**Table 1:** Participant inclusion and exclusion criteria

Inclusion criteria	Exclusion criteria
Age group between 18 and 80 years	Age <18
Undergoes endotracheal intubation	Patients who received sedatives or paralyzing agents prior to admission to the emergency department
Glasgow Coma Scale score ≥13	Glasgow Coma Scale score <13
Consenting for the study	Patients with limited mouth opening, trauma patients, especially facial trauma, cervical spine instability/immobility, anatomical airway abnormalities, and edentulous patients
Documentation done immediately prior to the administration of induction medications	Patients who expired prior to data collection

Data collection

The data collection form was designed to collect pertinent information about the study participants. This included data on patient demographic details, unique identifier numbers, and diagnoses. Data were collected on bedside airway predictors, including body mass index (BMI), retrognathia, the upper lip bite test (ULBT), thyromental distance (TMD), and hyomental distance (HMD). The accuracy of these parameters in predicting difficult airways was evaluated by comparing them against the classical MMC 3/4, which served as the reference standard in our diagnostic evaluation. All data assessments were performed by a single, trained emergency healthcare professional to minimize inter-observer variability.

Objectives

The study primarily aimed to develop an easy, innovative, and uncomplicated scoring system for predicting difficult airways. The secondary objectives were to evaluate the MMC to determine the incidence of difficult airways in the ED and to evaluate the sensitivity and specificity of the individual bedside predictors (BMI, retrognathia, ULBT, TMD, and HMD) against an MMC of 3/4. This study sought to elucidate the significance of evaluating airway conditions and enhancing collaborative care among interprofessional teams to improve patient outcomes in cases of airway compromise.

Definitions

BMI is a simple and non-invasive method to estimate whether a person falls within a healthy weight range based on height and weight. BMI is calculated by dividing a person's weight in kilograms by their height in meters squared. BMI is classified as underweight (<18.5 kg/m^2^), normal healthy individual (18.5-24.9 kg/m^2^), overweight (25.0-29.9 kg/m^2^), and obese/difficult airway (30.0-34.9 kg/m2) [12]. As BMI increases, the likelihood of encountering a difficult airway also increases [12]. Patients with a BMI >30 kg/m^2^ are assigned a score of one, while those with a BMI <30 kg/m^2^ receive a score of 0.

Retrognathia is a condition where the mandible is set back or underdeveloped relative to the maxilla, which can reduce the space required for easy intubation and is therefore considered a difficult airway predictor [13]. Retrognathia was assessed clinically by visual inspection of mandibular position relative to the maxilla in a neutral head position. Although retrognathia is commonly seen in syndromic patients, in this study, it was assessed as an independent anatomical predictor irrespective of the etiology. A score of one is given for patients with retrognathia, and a score of 0 is given for those patients who did not have retrognathia.

The ULBT is a critical bedside assessment used to predict difficult airway conditions. The ULBT is also described in literature as the Calder bite analysis system, which functionally reflects mandibular protrusion. It assesses the ability of the lower incisors to reach or cross the vermilion border of the upper lip. Patients who cannot achieve this have a notably increased chance of encountering difficulties during airway management or intubation. The capacity to bite the upper lip is grouped into three classifications: Class 1, where the patient can bring their lower teeth above the edge of the upper lip or vermilion border; Class 2, where the patient can bite their upper lip but only below the vermilion border; and Class 3, where the patient cannot bite their upper lip at all [14]. Patients classified as Class 3 (unable to bite their upper lip at all) were assigned a score of one, indicating potential difficulty, while patients who can perform the test (Class 1 or 2) were assigned a score of 0.

The TMD is the measurement from the chin to the top of the thyroid cartilage notch when the head is in its fully extended state, which is commonly used to assess potential airway difficulties. A TMD of less than 7 cm was considered indicative of potential difficulty. A shorter TMD suggests a higher risk of airway challenges. Typically, a normal TMD is considered to be greater than 7 cm [15]. Patients with a TMD of less than 7 cm are assigned a score of one, while those with a TMD greater than 7 cm are assigned a score of 0.

The HMD is the space between the hyoid bone and the tip of the chin. A short HMD serves as an indicator of limited head extension, suggesting potential difficulty during laryngoscopy. Based on the space, the HMD is classified as Grade 1 (>6 cm), Grade 2 (4-6 cm), and Grade 3 (<4 cm) [16]. For scoring simplicity and consistency with existing airway risk assessment literature, the HMD was dichotomized using a 6 cm threshold. Patients with an HMD of 4-6 cm (Grade 2) or <4 cm (Grade 3) were given a score of one. Those with an HMD of >6 cm (Grade 1) were given a score of 0.

The MMC is a tool used to assess how easily an endotracheal tube can be placed. The test visually examines the distance between the tongue's base and the mouth's roof. It is considered one of the most reliable non-invasive methods for evaluating the airway. MMC assessment was performed with the patient sitting (or semi-Fowler position) and instructed to maintain a neutral neck position with maximal mouth opening and tongue protrusion without phonation. The examiner then observes the oral cavity, noting the visibility of the uvula, faucial pillars, and soft palate. This scoring is done without asking the patient to make any sounds. The scores were classified as: Class 1 (MMC 1), where the faucial pillars, soft palate, and uvula are visible; Class 2 (MMC 2), where the faucial pillars and soft palate are visible, but the tongue hides part of the uvula; Class 3 (MMC 3), where only the soft palate is visible; and Class 4 (MMC 4), where only the hard palate is visible [8]. Both MMC 3 and 4 signify a restricted view of the oropharynx, suggesting an increased risk of difficult intubation; they are therefore categorized as indicative of a difficult airway and were given a score of one, while patients with MMC 1 and 2 were given a score of 0.

Methodology

This study, conducted from January 2023 to August 2025, is a prospective, observational, and comparative analysis performed in the ED of a tertiary medical center. All patients who satisfied the inclusion and exclusion criteria were included in the study. The study collected data on five predictors: BMI, retrognathia, ULBT, short TMD, and short HMD. Along with these parameters, the MMC was also analyzed for all study participants. The final scores were compared to the MMC score of 3/4 to evaluate their accuracy in predicting difficult airways. All airway assessments were performed by a single emergency healthcare professional to minimize inter-observer variability. The scoring system used is represented in Table 2.

**Table 2:** The novel scoring system used BMI, body mass index; ULBT, upper lip bite test; TMD, thyromental distance; HMD, hyomental distance

Predictor	Condition	Score
BMI	>30 kg/m²	1
Retrognathia	Present	1
ULBT	Unable	1
TMD	<7 cm	1
HMD	HMD of 4-6 cm (Grade 2) or <4 cm (Grade 3)	1

The scoring system has a lower score of 0 and an upper limit of five. A score greater than or equal to one is considered positive, while a score less than one is considered negative.

Statistical analysis

All statistical analyses were performed using IBM SPSS Statistics 20 (SPSS Inc., Chicago, USA). All continuous variables were presented as mean±SD, and all categorical variables were presented as frequencies with percentages. The McNemar chi-square test was applied to compare the findings of the new score with the reference standard MMC. The agreement was analyzed by the kappa coefficient. Diagnostic parameters like sensitivity, specificity, positive predictive value (PPV), negative predictive value (NPV), and accuracy were estimated in percentages with a 95% CI. A p-value of <0.05 was considered statistically significant. All statistical tests were two-tailed. ROC analysis was performed using SPSS software.

## Results

Based on the inclusion and exclusion criteria, a total of 200 participants (n=200) were recruited. Among the study sample, the majority of the population comprised males (n=114, 57%) rather than female participants (n=86, 43%). The mean age of participants was 52.5±20.5 years. Most participants presented to the ED with abdominal pain (n=13, 6.5%), fever (n=12, 6%), breathlessness (n=10, 5%), stroke (n=9, 4.5%), and vomiting (n=6, 3%). The contingency table using the parameter BMI >30 kg/m² for evaluating difficult airways against the MMC is represented in Table 3.

**Table 3:** Diagnostic accuracy of the parameter BMI >30 kg/m² for predicting difficult airways (using the Modified Mallampati classification as the reference standard) BMI, body mass index; MMC, modified Mallampati classification; TP, true positives; FP, false positives; FN, false negatives; TN, true negatives

BMI >30 Kg/m² prediction	MMC: difficult (3,4)	MMC: easy (1,2)
Positive (parameter present)	39 (TP)	9 (FP)
Negative (parameter absent)	43 (FN)	109 (TN)
Metrics	Sensitivity: 47.56% (39/82)	Specificity: 92.37% (109/118)

The numbers of true positives (TP), false positives (FP), false negatives (FN), and true negatives (TN) were 39, 9, 43, and 109, respectively. It had a sensitivity of 47.56% (39/82) and a specificity of 92.37% (109/118). The results obtained using the parameter retrognathia for evaluating difficult airways against the MMC are represented in Table 4.

**Table 4:** Diagnostic accuracy of the parameter retrognathia for predicting difficult airways (using the MMC as the reference standard) MMC, modified Mallampati classification; TP, true positives; FP, false positives; FN, false negatives; TN, true negatives

Retrognathia prediction	MMC: difficult (3,4)	MMC: easy (1,2)
Positive (parameter present)	17 (TP)	11 (FP)
Negative (parameter absent)	65 (FN)	107 (TN)
Metrics	Sensitivity: 20.73% (17/82)	Specificity: 90.68% (107/118)

The numbers of TP, FP, FN, and TN were 17, 11, 65, and 107, respectively. It had a sensitivity of 20.73% (17/82) and a specificity of 90.68% (107/118). The results obtained using the parameter ULBT for evaluating difficult airways against the MMC are represented in Table 5.

**Table 5:** Diagnostic accuracy of the parameter ULBT for predicting difficult airways (using the MMC as the reference standard) ULBT, upper lip bite test, MMC, modified Mallampati classification; TP, true positives; FP, false positives; FN, false negatives; TN, true negatives

ULBT prediction	MMC: difficult (3,4)	MMC: easy (1,2)
Positive (parameter present)	17 (TP)	6 (FP)
Negative (parameter absent)	65 (FN)	112 (TN)
Metrics	Sensitivity: 20.73% (17/82)	Specificity: 94.92% (112/118)

The numbers of TP, FP, FN, and TN were 17, 6, 65, and 112, respectively. It had a sensitivity of 20.73% (17/82) and a specificity of 94.92% (112/118). The results obtained using the parameter short TMD for evaluating difficult airways against the MMC are represented in Table 6.

**Table 6:** Diagnostic accuracy of the parameter short TMD for predicting difficult airways (using the MMC as the reference standard) TMD, thyromental distance; MMC, modified Mallampati classification; TP, true positives; FP, false positives; FN, false negatives; TN, true negatives

Short TMD prediction	MMC: difficult (3,4)	MMC: easy (1,2)
Positive (parameter present)	40 (TP)	19 (FP)
Negative (parameter absent)	42 (FN)	99 (TN)
Metrics	Sensitivity: 48.78% (40/82)	Specificity: 83.90% (99/118)

The numbers of TP, FP, FN, and TN were 40, 19, 42, and 99, respectively. It had a sensitivity of 48.78% (40/82) and a specificity of 83.90% (99/118). The results obtained using the parameter short HMD for evaluating difficult airways against the MMC are represented in Table 7.

**Table 7:** Diagnostic accuracy of the parameter short HMD for predicting difficult airways (using the MMC as the reference standard) HMD, hyomental distance, MMC, modified Mallampati classification; TP, true positives; FP, false positives; FN, false negatives; TN, true negatives

Short HMD prediction	MMC: difficult (3,4)	MMC: easy (1,2)
Positive (parameter present)	39 (TP)	17 (FP)
Negative (parameter absent)	43 (FN)	101 (TN)
Metrics	Sensitivity: 47.56% (39/82)	Specificity: 85.59% (101/118)

The numbers of TP, FP, FN, and TN were 40, 19, 42, and 99, respectively. It had a sensitivity of 48.78% (40/82) and a specificity of 83.90% (99/118).

The novel scoring system exhibited strong diagnostic performance. It achieved a high sensitivity of 79.27%, indicating its robust ability to correctly identify patients with a difficult airway. The score's specificity was 71.19%, demonstrating its effectiveness in correctly classifying patients with an easy airway. Furthermore, the PPV was 65.66%, suggesting that a positive score correctly identified a difficult airway in approximately two-thirds of cases. The high NPV of 83.17% indicates that a negative result from the new score is a highly reliable predictor of an easy airway. Overall, the new scoring system demonstrated a diagnostic accuracy of 74.50% (Table 8).

**Table 8:** Predictive accuracy of the new airway score relative to the MMC MMC, modified Mallampati classification; TP, true positives; FP, false positives; FN, false negatives; TN, true negatives

New airway score	MMC	Outcome type	n	Percentage (%)
Positive (≥1)	Difficult (3-4)	TP	65	32.5
Positive (≥1)	Easy (1-2)	FP	34	17.0
Negative (0)	Difficult (3-4)	FN	17	8.5
Negative (0)	Easy (1-2)	TN	84	42.0
Total	Difficult (3-4)	-	82	41.0
Total	Easy (1-2)	-	118	59.0
Grand total	-	-	200	100

**Figure 1:**
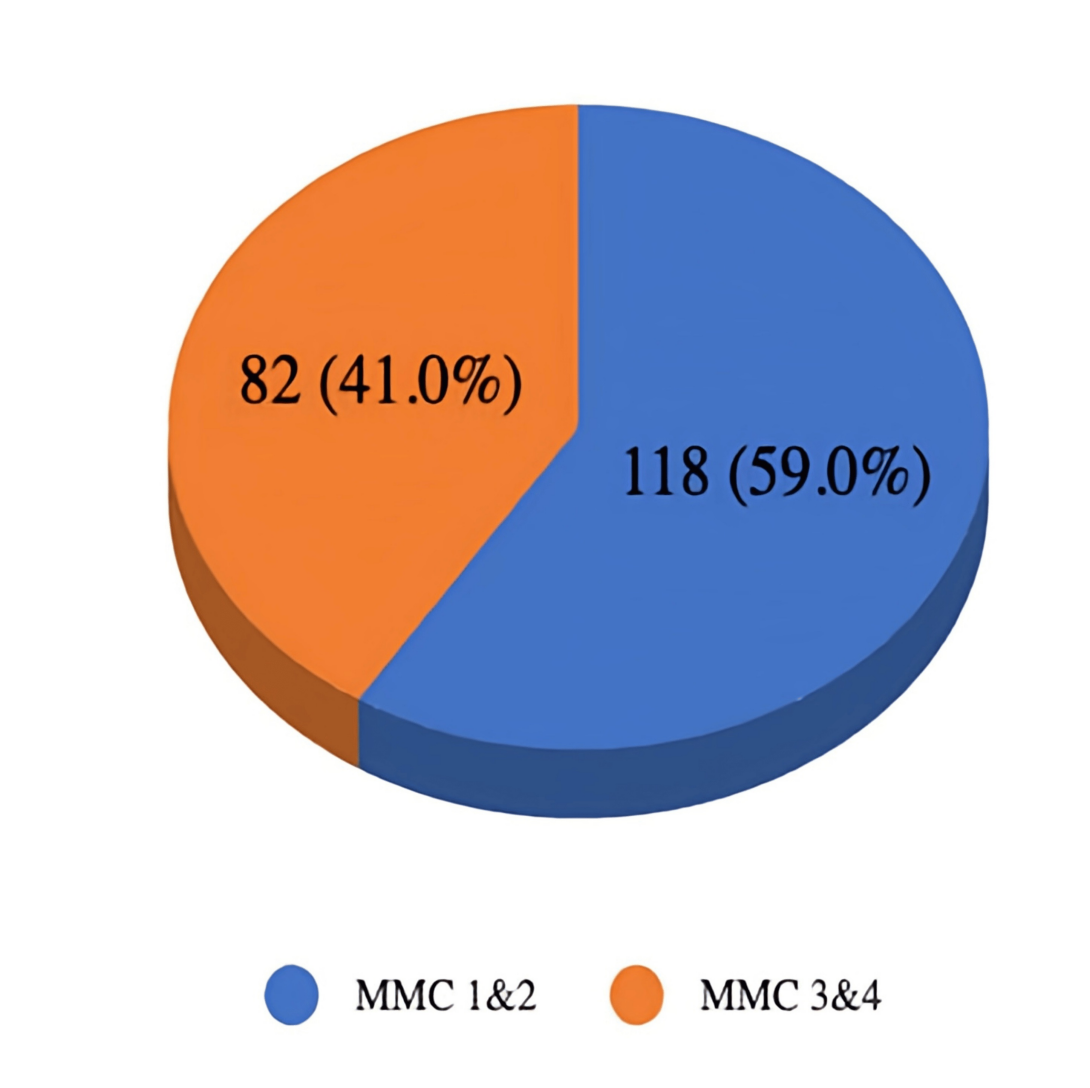
Graphical representation of the incidence of difficult airways MMC, modified Mallampati classification

The relationship between the new scoring system and the MMC was further analyzed using statistical tests. The McNemar chi-square test revealed a statistically significant difference between the two methods (χ²=5.67, df=1, p=0.017) with an effect size of Cohen's g=0.085. This indicates that the new score's classification of patients as difficult or easy airways is not identical to that of the MMC. Furthermore, the agreement between the two scores was assessed using the kappa coefficient, which was calculated to be 0.489. This value signifies a moderate level of agreement, and the finding was statistically significant (p<0.001). The incidence of difficult airways is represented in Figure 1. Among the 200 patients, 82 had an MMC score of three or four; thus, 41% of the population experienced a difficult airway.

**Figure 2:**
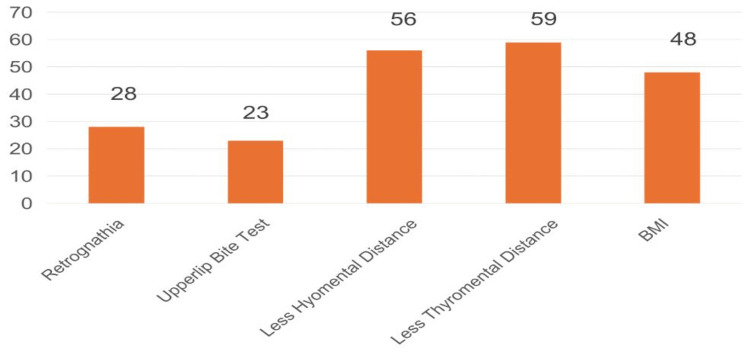
Frequency of the individual predictors in the new scoring system BMI, body mass index

The frequency of the individual predictors is represented in Figure 2.

Among the five predictors, short TMD had the highest frequency (n=59), and the inability to perform an ULBT had the lowest frequency (n=23).

Receiver operating characteristic (ROC) curves were drawn, demonstrating a clear deviation from the reference (diagonal) line and indicating strong discriminative ability. The AUC was 0.752 (SE=0.036, 95% CI: 0.683-0.822, p<0.001), which denotes good overall diagnostic accuracy. An integer cutoff score of one showed the highest sensitivity (79.3%) and specificity (71.2%), with the highest Youden index (50.5%) among other scores, as represented in Table 9.

**Table 9:** Sensitivity, specificity, and Youden index of the new five-parameter-based scoring system

Score cut-off (≥)	Sensitivity (%)	1 − Specificity (%)	Specificity (%)	Youden’s Index (%)
1	79.3	28.8	71.2	50.5
2	62.2	19.5	80.5	42.7
3	36.6	3.4	96.6	33.2
4	7.3	0.8	99.2	6.5
5	0	0	100	0

**Figure 3:**
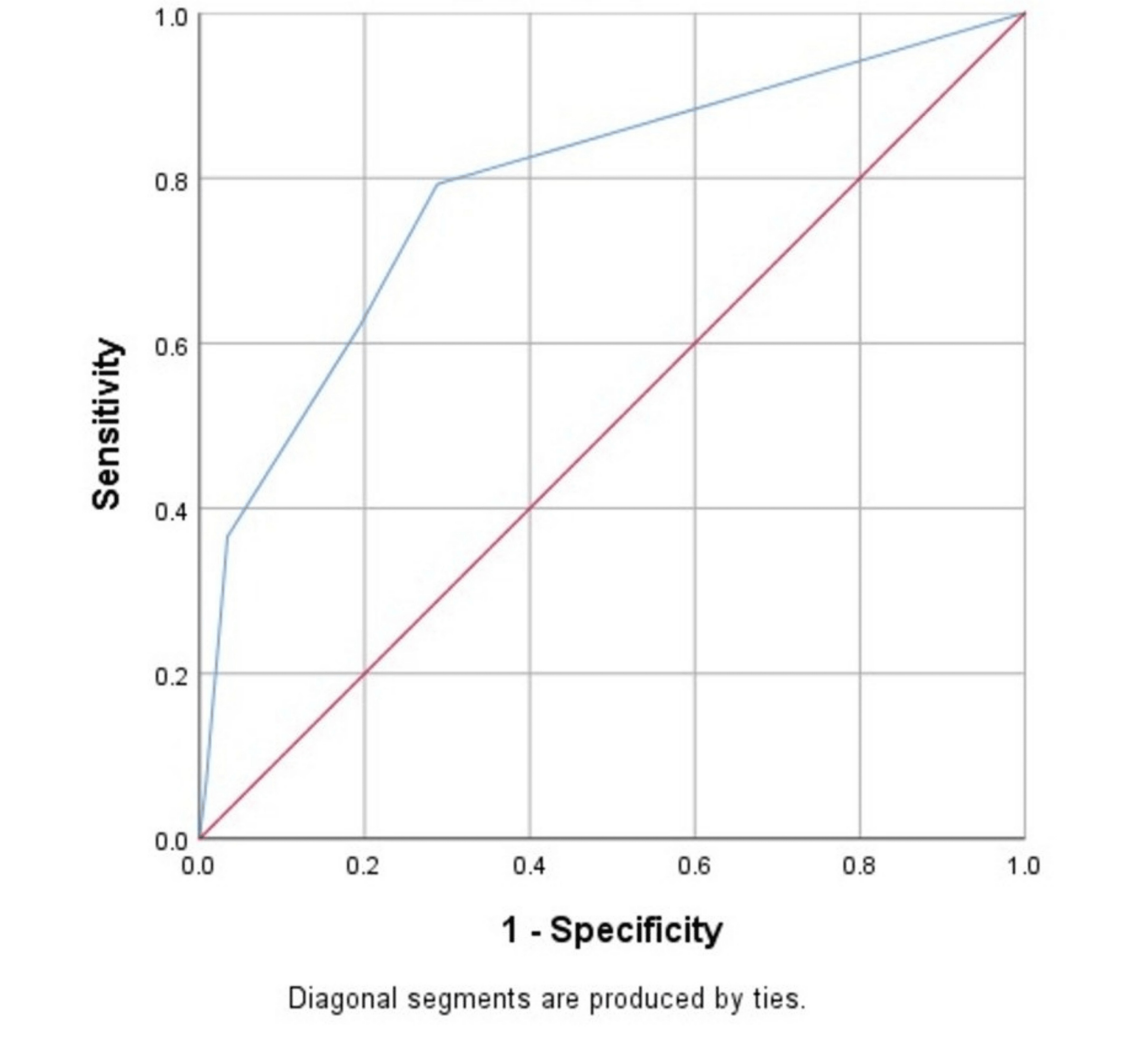
ROC curve depicting the diagnostic performance of the novel five-parameter bedside score compared with the MMC ROC, receiver operating characteristic; MMC, modified Mallampati classification

Figure 3 illustrates the ROC curve, which demonstrates a clear deviation from the reference (diagonal) line, indicating strong discriminative ability. The AUC was 0.752 (SE=0.036, 95% CI: 0.683-0.822, p<0.001), which denotes good overall diagnostic accuracy.

## Discussion

The diagnostic accuracy of our new composite score holds significant clinical value for risk stratification and resource management in the ED. The PPV of 65.66% means that when the score predicts a difficult airway, approximately 66 out of every 100 predictions will genuinely require advanced airway management. While this includes some false positives, this approach ensures preparedness, which is essential for patient safety. The NPV of 83.17% provides strong confidence that when the score predicts an easy airway, approximately 83 out of every 100 patients will be suitable for routine intubation protocols, allowing for resource optimization in the busy ED. The score functions as a necessary high-sensitivity screening tool, ensuring that few difficult cases are missed (FN=17), thereby reducing the incidence of unanticipated difficult intubations, which are directly linked to critical complications like hypoxemia.

The McNemar test revealed a statistically significant difference (p=0.006) in the proportions of discordant pairs between the new score and the MMC. This finding indicates that a statistically significant number of patients were classified differently by the two methods, suggesting that the classifications are not interchangeable on a patient-by-patient basis. This significant difference highlights that the new score offers a distinct pattern of classification, particularly in how it shifts patients from "easy" to "difficult" compared to the MMC alone, which could lead to different and potentially safer clinical decisions.

Our findings are consistent with those of Liao et al., who highlighted the limitations of the MMC in emergency intubation scenarios [11]. However, our study advances the field by integrating multiple predictors, resulting in a more accurate and dependable assessment tool. While individual predictors like retrognathia are universally recognized in the literature (e.g., 17), their systematic integration and validated contribution within concise, multi-parameter, ED-specific scores, unlike factors such as BMI in other scores (e.g., 223), remains an area requiring further investigation, which our study addresses by rigorously evaluating its role.

While individual predictors, such as the ULBT, demonstrated very high specificity (94.92%), their isolated use would result in an unacceptable number of missed difficult airways due to low sensitivity (20.73%). The purpose of the new score is not to maximize the specificity of any single factor, but rather to optimize overall accuracy by balancing sensitivity and specificity. In the ED context, we prioritized sensitivity (79.27%) to reduce the risk of life-threatening FN. The resulting aggregate specificity of 71.19% reflects this necessary trade-off for a multi-parameter model, as the score must cast a wider net to prevent catastrophic CICO situations. Our results show that the scoring system we developed has a sensitivity of 79.2%, making it a promising tool for predicting difficult airways. In comparison to the classic Mallampati classification, the predictors of retrognathia and the ULBT showed a greater specificity, suggesting that these predictors are more effective in identifying cases of difficult intubation.

This AUC value substantiates the balance between sensitivity (79.27%) and specificity (71.19%) observed in the individual diagnostic indices. The score, therefore, exhibits a strong capability in distinguishing between patients with and without a difficult airway. The ROC characteristics highlight the model’s effectiveness as a high-sensitivity screening tool, ensuring that potentially difficult cases are reliably identified in the ED setting.

In the present study, we examined five specific predictors of a difficult airway: BMI, retrognathia, ULBT, TMD, and HMD. For instance, tests such as the ULBT and retrognathia did not consistently identify all difficult airways (indicating lower sensitivities). However, when these tests indicated the presence of a difficult airway, they were highly accurate (indicating high specificities), making them valuable tools for confirming the diagnosis [17]. Existing literature also supports the clinical utility of these individual assessments. By integrating these various tests into a single scoring system, our approach provides a more comprehensive assessment and mitigates the limitations of depending on any one test alone.

The rationale behind developing a new combined score stems from the limitations of traditional methods, such as the Mallampati classification. The Mallampati classification depends on visual inspection and can be subjective, thereby reducing its reliability, particularly in a busy ED or with uncooperative patients [18]. The consensus in the medical literature is that no single test can accurately predict a difficult airway, which is why multi-parameter scoring systems like LEMON and SARI are commonly employed. Our newly developed score aligns with this approach by integrating multiple factors to provide a more comprehensive assessment.

This simple score plays a crucial role in enhancing patient safety in the ED. By promptly identifying patients at risk for a difficult airway, emergency teams can be better prepared, thereby minimizing intubation attempts and preventing serious complications such as hypoxia or cardiac arrest. This proactive strategy helps to avoid critical situations, including CICO scenarios [4]. The simple, five-parameter format of this score significantly streamlines the assessment process, allowing for easy integration into simulation-based training modules for emergency healthcare professionals, thereby reducing the learning curve and accelerating proficiency in difficult airway prediction.

Limitations

The major limitation of the present study was that we did not include the Cormack-Lehane classification. We used the MMC as the reference standard for predicting airway difficulty, compared the MMC with five airway predictors, and formulated a validated score. In this study, because ULBT and BMI assessments require patient cooperation, unconscious patients were excluded. Therefore, future research will focus on validating the score in unconscious patients and will compare these five predictors with both the MMC and Cormack-Lehane classification for validation.

While the Mallampati classification is widely used, the Cormack-Lehane classification, which assesses the actual view during intubation, is considered a more definitive measure of difficulty. In future studies, we plan to validate our score against the Cormack-Lehane classification to further confirm its accuracy and usefulness in real-world intubation scenarios. The airway assessments were conducted by a single, trained investigator. While this ensured consistency in the application of the novel score, the lack of double-blinding may introduce potential observer bias. Double-blinding was not feasible due to the nature of bedside anatomical airway assessments in emergency settings. Our study included all conscious adult patients presenting to the ED who required intubation, regardless of their pre-assessment for a difficult airway. Further research is recommended to validate these findings in different patient groups.

## Conclusions

This prospective study introduces a preliminary validation of a simple, non-invasive, five-parameter bedside score that demonstrated a strong discriminatory ability in predicting difficult airways in the ED. With good sensitivity, specificity, and AUC values, this score outperforms reliance on single predictors and provides a more robust assessment than the MMC alone. The use of rapidly obtainable anatomical and clinical parameters enables early risk identification, supports proactive airway planning, and may reduce the incidence of unanticipated difficult intubations. Its high NPV offers emergency healthcare professionals the confidence to exclude a difficult airway, thereby reducing unnecessary advanced preparations and optimizing resource allocation in the busy ED environment. Practical, quick to perform, and easily applicable at the bedside, this score serves as an effective decision-support tool that enhances patient safety and improves clinical efficiency and cost-effectiveness. Further multicenter validation, including evaluation in unconscious or sedated patients and correlation with Cormack-Lehane laryngoscopic views, is warranted to expand its generalizability and confirm broader clinical relevance.
